# Treatment of Dysentery

**Published:** 1851-07

**Authors:** J. Watson Tullis

**Affiliations:** Troy, Ohio


### ARTICLE V.

*Treatment of Dysentery.* By J. WATSON TULLIS, M. D. OF TROY, OHIO.

Dysentery prevailed very extensively in Troy and its vicinity, during the latter part of the summer of 1850. It was marked by the usual symptoms of epidemic dysentery. A number of cases proved fatal, under the ordinary treatment. I mean by ordinary, the mercurial and anodyne treatment, with the occasional use of cathartics.

During previous years, I had treated dysentery according to the plan generally recommended in the books, but never with the uniformly happy result which attended an almost entirely different course of treatment, adopted by me during the last summer.

I had lost confidence in the mercurial treatmeut, not only from my own experience, but from the numerous concessions as to its uncertainty, on the part of those who had trusted in it.

The cathartic treatment, resorted to by almost all, so far as I know, it seems to me, is still more objectionable. Although it induces fœcal evacuations, yet it does so at the expense of the delicate and inflamed mucous membrane of the bowels. The natural evacuations, thus induced, do not depend on a subsidence of the *disease*, but upon the excited contractions of the muscular coat of the bowels, upon the already inflamed and painful mucous membrane, which must inevitably aggravate the pain and inflammation, and consequently increase the danger of the case. If the mucus secreted is lessened or suppressed by such treatment, it is because there is such an exaltation or organic action, as is incompatible with the process of secretion; and the mucus, or muco-bloody discharges will return, probably with increased profusion and frequency; unless, indeed, from the mildness of the disease, or the recuperative power of the system, recovery is effected in spite of the treatment.

But I will briefly present the simple treatment which I have adopted, and which I can recommend, with the utmost confidence, to my medical brethren.

1 direct sulphate of morphine, in doses varying from 1-6 to 1-3 of a grain, one of which is to be taken every time the bowels are moved, provided the interval be not less than one-half hour; thus giving time for the action of the medicine, lest the patient be narcotized. I uniformly give 10 or 12 or more powders, of quantity suited to the patient. If I know nothing of the susceptibility of the patient to the action of morphine, I sometimes direct that he should begin with half-powders, repeating as above; but if not very soon relieved, to take full doses.

I never used morphine in such profusion as in our late epidemic, and yet, in an extensive practice did not narcotiz a single patient. It is astonishing what an amount of morphine, in dysentary as well as in many other diseases, is tolerated by those who, under ordinary circumstances, would be readily narcotized.

Having adopted the morphine treatment during the entire period of this epidemic, and in a variety of constitutions and with, certainly, not less complications than are usually met with in dysentery, during the summer and fall months, I am inclined to prefer it very much, above any other treatment. It is safe, salutary, and free from the horrible torture incident to the mercurial and cathartic treatment.

Of course, regimen is of the utmost importance in this affection. Total abstinence from food, mucilaginous drinks, and a suitable dose of morphine after each evacuation of the bowels, will frequently effect a permanent cure, in less than twenty-four hours.

It was only in a few cases necessary to use enemas of laudanum and starch. This, however, should never be neglected, if tenesmus and tormina resist the morphine.

In all cases which resisted treatment by internal means, or were marked by unusual pain, or tenderness of the abdomen, or great prostration, or any other indication of danger, I cupped *freely* and *repeatedly* and with the *very happiest effect.* In an acute case of dysentery, not controllable by ordinary means the neglect of local depletion is unpardonable. Perhaps the only cases which would not recover without medical aid, are left to perish for the want of *local bleeding*, under the delusion that a *blister* is as salutaey as *sacrificator* and *cups.* This delusion or opinion, is not sanctioned by any modern work extant; and yet, perhaps, more than nine-tenths of our western country practitioners, practice upon it, to the torture and sacrifice of hundreds of patients.

Warm emolient poultices, constantly applied, are of unquestionable utility, and should never be neglected in serious cases.

Blisters should never be applied in acute dysentery, because they are a thousand times more painful than cupping, and a thousand times less useful, and because when they fail, you can neither repeat the blister, nor apply cups, until the surface be healed.

In conclusion, permit me to say that, in all ordinary cases of dysentery, suitable doses of morphine, taken immediately after each dejection, with rest and abstinence from food, will effect a speedy cure. As to the severer cases of dysentery, I propose to make further observations in a subsequent article.—*Ohio Med. and Surg. Journal.*

